# Concurrent Opioid and Psychotropic Drug Use During Pregnancy: Descriptive Study from Two Canadian Provinces

**DOI:** 10.23889/ijpds.v9i5.2857

**Published:** 2024-09-18

**Authors:** Lindsey Dahl, Deepa Singal, Marni Brownell, Colin Dormuth, Dan Chateau, Matthew Dahl, Jason Kim, Nathan Nickel

**Affiliations:** 1 Manitoba Centre for Health Policy; 2 University of British Columbia; 3 Australian National University

## Objective

This study aims to describe the patterns of concurrent opioid and psychotropic drug use (CU) during pregnancy and the characteristics of the mothers dispensed these drugs.

## Methods

Pregnancies between 2000-2020 were identified using administrative data from Manitoba and British Columbia, Canada. Prenatal opioid and psychotropic drug dispensations were assessed, and exposure groups created based on the number of dispensations within each drug category. Descriptive statistics are reported.

## Results

Approximately 1.4% of 1,040,021 pregnancies involved CU. Odds of CU were greater for 2+ opioid pregnancies compared to pregnancies with only 1 opioid (OR=4.05). Among CU pregnancies, it was more likely for 2+ types of psychotropics to be dispensed for those involving 2+ opioids compared to 1 (OR=2.23). Percent of CU pregnancies did not change over time, while opioid pregnancies decreased, and psychotropic pregnancies increased. Average morphine equivalent dose of opioids/pregnancy was higher in CU than opioid only pregnancies. Compared to mothers without exposure, CU exposed mothers were of similar age, more likely to live rurally, in lower income areas, and have lower care continuity.

## Conclusion

CU pregnancies remained stable over time. It was more likely for mothers dispensed 2+ opioids to be CU than mothers dispensed only 1 opioid, and more likely for CU mothers dispensed 2+ opioids to be dispensed 2+ psychotropic types. Overall strength of opioids was higher for CU mothers compared to opioid only exposed mothers.

## Implications

Studies measuring the impact of CU on maternal and child outcomes should be conducted to inform safe prescribing practices.

